# The Association of Military Surgeons of the United States—A Question of Statistics

**Published:** 1905-10

**Authors:** 


### The Association of Military Surgeons of the United States—A Question of Statistics.

THE fourteenth annual meeting of the Association of Military Surgeons at Detroit, September 25 to 29, 1905, was one of the most successful ever held, and by reason of a statistical discussion, attracted more newspaper attention than usual. The first few days of the session were marked by the presentation of papers remarkable for their excellence and the high type of discussion which was participated in by medical officers from Germany, Japan, China, England, Mexico, Canada and France, as well as surgeons of the United States army, navy, and the National Guard.

The sensational feature of the meeting was the attack by Dr. Charles F. Stokes, of the navy, on a paper read at the previous meeting of the association at St. Louis, by Dr. Louis L. Seaman, which dealt with the medical department of Japan and its work during the Russo-Japanese war. Dr. Stokes, after reading a prepared paper, asked permission to reply to Dr. Seaman's St. Louis paper. After referring to the latter's statistics of Japanese origin, he said:

> Not only have these misleading statements and erroneous assertions done serious injury and great injustice to the military surgeons of this country and this association, but they have jeopardised the reputation of Dr. Seaman as a military surgeon and as a sanitarian.
>
> His statistical report of the Spanish-American and the Civil War, are at variance with the official records.
>
> Dr. Seaman stated that in a campaign lasting six weeks the proportional casualty in the Spanish-American war was one death in action to fourteen from disease. But the report of the Secretary of War for 1898 gives the following: Killed and died of wounds, 270; died from disease, 400.
>
> Dr. Seaman makes the latter 3,862. The proportion of battle casualties to deaths from disease was, according to official report, one to one and one-half. This includes the losses in the fifth army corps from its organisation at Tampa to its disbandonment at Montauk.
>
> The doctor says concerning sickness in Oku's army that only 40 out of 5,609 soldiers sent home, died.
>
> He must have been in error there. Forty cases of sudden death did occur at the front, but all the cases of serious illness were at once transferred to the base hospital in the rear.

Of the total of 5,609 cases there were 5,070 cases of beriberi, a disease with a mortality almost as high as typhoid fever.

In the second Japanese army of three divisions with 19,000 men in each, there was a sick list of 24,642 in seven months, which gives an average mortality rate of 740 per 1,000, not very different from that in other armies under favorable conditions.

We see none of these things in Dr. Seaman's reports. Beriberi is almost unknown there," is the statement made by Dr. Seaman before the military affairs committee of the House.

Now the medical statistics are not out yet, but it is known that the first Japanese army had in four months 4,609 cases of beriberi, and that the second in seven months had 5,070, and it is further said that the loss to the effective force of General Nogi's army before Port Arthur was 25,000 from this cause.

Dr. Seaman did not answer until the next day when he read his paper on "The real triumph of Japan, or the conquest of the silent foe." He then made clear his position and showed by reference to official reports and quotations from his St. Louis address that Dr. Stokes had woefully misunderstood the statistics. He showed that his St. Louis paper contained the statement that the Japanese navy had conquered beriberi by a change in ration; "that the army is less fortunate. When I left Newchang in August kakki began showing itself in the ranks;" and he then stated that unless the army ration was changed beriberi would continue. In the August following the ration was changed and beriberi decreased steadily.

Concerning Dr. Stokes's statement that Dr. Seaman had spoken of a campaign of "six weeks" in the Spanish war, Dr. Seaman by reference to printed reports showed that his statement read: "In a campaign, the actual hostilities of which lasted only six weeks," and proceeded to a stinging rebuke for the apparent emasculation of his statements. Dr. Stokes's statement that there were 270 deaths from wounds and only 400 from disease during the Spanish war was disproved by Seaman, whose figures showed deaths from wounds 268, and from disease 3,862, or about 14 to 1; and he also showed that his figures were correct as it is possible to have disease statistics; in fact his answer to the statements by Dr. Stokes was convincing and absolute.

The mere fact of a disagreement as regards the simple statistical phase of the question rather tended to cloud the main issue. Dr. Stokes and Dr. Seaman were deeply in earnest in their charges and replies; they verged toward bitterness and it was evident that each was laboring under more or less excitement during the periods they held the floor, especially when Dr. Stokes stated that he had high regard for the Japanese surgeons, but he "could not allow the standing of the medical men of the United States army to be called into question without answer."

It is apparent that in that statement lies the chief reason for the attack on Dr. Seaman. It showed the exquisite tenderness of the army and navy commissioned officer regarding anything affecting their service, however remote or suggestive. In such an event there is a call to arms in defense of their personal pride and with eyes shut to their own interests they spring to reply. Dr. Seaman has not yet made any statement detrimental to the medical department of either the navy or the army; on the contrary, in all his statements which have been published, including his evidence before the House military committee last year, he spoke in the highest terms of the personnel of the medical corps. But always and persistently he has fought for a betterment of the service and an increase in the powers of the medical officer; he asks recognition for the head of the department and an increase in the numerical strength of the corps. He has drawn these uncomplimentary statistical comparisons, not with any view to belittling the ability of the American surgeon and the aggrandisement of the Japanese surgeon, but with a view to showing that the Japanese service is better because its officers have more power to enforce their orders regarding sanitation, medication, and feeding an army.

Were Dr. Seaman of the opinion that the army medical department was inefficient he would hardly have recommended to the House committee that the surgeon-general be raised to the rank of a major-general responsible only to the Secretary of War and the President; and the thinking profession must agree with him that the medical department of the United States army is at the mercy of the general staff, a body of short-sighted, unscientific men who deal only with the visible engines of destruction and know absolutely nothing of the silent foe which was conquered by the Japanese surgeons, because they had the power and the rank and the authority to issue orders of sanitation and compel their enforcement. Dr. Stokes and the commissioned officers of the army and navy may consider themselves aggrieved by Dr. Seaman's statements and statistics; yet the fact remains that the statistics brought forth in reply to Dr. Seaman's St. Louis paper were misleading, if not juggled; for Dr. Stokes's statement that there were only 400 deaths from disease during the Spanish war brought reply from Dr. Jefferson D. Griffith, surgeon-general of Mississippi, who was in charge of a brigade at Chicamauga, that there were under his own observation at that delectable camp 600 deaths from typhoid alone.

It is regrettable that the army and navy should seek to belittle Dr. Seaman's efforts in the direction of the betterment of their corps. One cannot hope to get anything from Congress without making out a case; and to do that one must "deliver the goods,"

to use a political colloquism, common under such circumstances; and Dr. Seaman has delivered them in such able fashion that were there any question regarding the necessity for a betterment of the medical department of the army, not in efficiency, but in numbers, it must have been swept aside and the way made clear for an increase in the corps.

In whatever quibbling may arise, and in all probability there will be much, the fact must not be lost sight of that we are not, as a profession, fighting for higher rank and more authority for the medical department alone, but primarily for the betterment of the condition of the enlisted man of the army; the man who does the actual fighting and carries the flag to conquest. His physical condition is at stake. No soldier can do duty with an empty stomach or a sick body, and regrettable though it is, in these days of modernity,that was the condition of the enlisted man during the Spanish war on many and many a day; days when racked with fever he appealed to his surgeon for relief and got—nothing; because the medical supplies were stopped at Tampa in order that mules might be shipped to the scene of action. That is what Dr. Seaman is fighting for and it would be in better taste if the commissioned officers of the army and navy would second his efforts instead of attempting to belittle or discredit his statements, in both of which Dr. Stokes so signally failed to do at the Detroit meeting.

Since the above was written Japan has issued an official statement showing that there was a total mortality in the period covered by the war in round numbers of 73,000, of which 15,000 died of disease, the balance of wounds; or over 3 deaths from bullets to 1 of disease, corroborating Dr. Seaman and reversing the usual order.

